# Development and Optimization of Irinotecan-Loaded PCL Nanoparticles and Their Cytotoxicity against Primary High-Grade Glioma Cells

**DOI:** 10.3390/pharmaceutics13040541

**Published:** 2021-04-13

**Authors:** Basant Salah Mahmoud, Christopher McConville

**Affiliations:** 1 College of Medical and Dental Sciences, School of Pharmacy, University of Birmingham, Birmingham B15 2TT, UK; BSM465@student.bham.ac.uk; 2 Hormones Department, Medical Research Division, National Research Centre, El Buhouth St., Dokki, Cairo 12622, Egypt

**Keywords:** nanoparticles, high-grade gliomas, polycaprolactone, irinotecan, emulsification, solvent evaporation, amorphization, electrolytes

## Abstract

Background: High-grade gliomas (HGGs) are highly malignant tumors with a poor survival rate. The inability of free drugs to cross the blood–brain barrier and their off-target accumulation result in dose-limiting side effects. This study aimed at enhancing the encapsulation efficiency (EE) of irinotecan hydrochloride trihydrate (IRH) within polycaprolactone (PCL) nanoparticles with optimized size and charge. Materials and Methods: IRH-loaded PCL nanoparticles were formulated using either the single emulsion (O/W, W/O and O/O) or double emulsion (W/O/O and W/O/W) solvent evaporation techniques. The nanoparticles were characterized for their size, zeta potential and EE, with the optimized nanoparticles being characterized for their drug release and cytotoxicity. Results: The amorphization of PCL and the addition of electrolytes to the aqueous phases of the W/O/W emulsion produced spherical nanoparticles with a mean diameter of 202.1 ± 2.0 nm and an EE of 65.0%. The IRH-loaded nanoparticles exhibited zero-order release and were cytotoxic against primary HGG cells. Conclusion: The amorphization of PCL improves its EE of hydrophilic drugs, while the addition of electrolytes to the aqueous phases of the W/O/W emulsion enhances their EE further. IRH-loaded PCL nanoparticles have the potential to deliver cytotoxic levels of IRH over a sustained period of time, enhancing the cell death of HGGs.

## 1. Introduction

Malignant brain tumors are associated with high mortality and morbidity rates, owing to the lack of long-term disease control. The majority of malignant brain tumors that affect adults are gliomas, with high-grade gliomas (HGGs) accounting for 75% [1]. For each 100,000 population, 3 to 5 persons develop gliomas annually, with a higher frequency in men. Although the fifth and sixth decades of life are the most prominent for glioma incidence, gliomas can develop at any age [2]. In 2016, the WHO included genetic criteria in classifying gliomas, which resulted in identifying anaplastic astrocytoma, anaplastic oligodendroglioma and mixed anaplastic oligoastrocytoma (grade III) and glioblastoma (grade IV) as high-grade gliomas, accounting for the majority of cases, with a 60 to 70% incidence rate [3]. Several hurdles limit the efficacy of glioma treatment: (1) glioma cells can metastasize to other tissues in the brain and their irregular margins make complete resection difficult; (2) the blood–brain barrier (BBB) prevents drug molecules from entering the brain at therapeutic concentrations [4]; (3) cancer stem cells contribute to tumor initiation and therapeutic resistance and (4) glioma cells are immortalized in nature and any cells left behind during surgery will develop into a new tumor [5].

The standard treatment for gliomas is the Stupp protocol, in which patients undergo surgical resection, followed by radiotherapy and subsequent treatment with temozolomide. This treatment protocol has been shown to increase survival to 14.6 months, compared to 12.1 months with radiotherapy alone [6]. Other treatment options include Gliadel^®^ wafers made of biodegradable polycarboxyphenoxy propane containing the chemotherapeutic drug Carmustine (Bis-chloroethylnitrosourea). However, a Cochrane report demonstrated that the Gliadel wafers provided no significant clinical benefit for newly diagnosed HGG patients, with a small but significant clinical benefit to recurrent HGG patients [7]. Furthermore, the Gliadel wafers suffer from poor drug penetration into the brain parenchyma, which means that the drug does not reach the deep-seated tumor tissue and reoperation is needed to implant additional wafers once they have finished releasing the drug, which introduces further invasiveness to the patient [8,9,10,11]. The problem with alkylating agents such as temozolomide is the presence of methyl guanine methyl transferase (MGMT), which can counteract their mechanism of action, leading to multidrug resistance (MDR). Therefore, there has been some research into a second line of drugs such as Carboplatin, Oxaliplatin and Irinotecan hydrochloride trihydrate (IRH). However, the systemic delivery of these chemotherapeutic drugs results in serious unwanted side effects for the patient, such as vomiting, diarrhoea, hair loss, neutropenia, myelosuppression and pain [12]. Traditional chemotherapeutic drugs also have poor in vivo stability, with off-target tissue accumulation, low bioavailability and an inability to cross the BBB, leading to larger doses being administered in order to achieve the therapeutic concentrations needed in the brain, resulting in the deterioration of the patient’s overall health.

Encapsulating drugs within nanoparticles (NPs) can offer a wide range of benefits that overcome some of the issues of traditional chemotherapy and could help to improve cancer treatment [13]. Drug-loaded NPs can cross the BBB by passive diffusion and accumulate in the tumor via the enhanced permeability and retention (EPR) effect, where they release their drug in a sustained fashion by diffusion and biodegradation [14]. NPs can protect drugs against early metabolism and reduce off-target tissue accumulation, which in turn increases treatment efficacy and protects healthy tissues. Drug encapsulation can extend the half-life of drugs and reduce the frequency of administration [14]. Targeting NPs by surface coating of antigen-specific antibodies can enhance the endocytosis uptake of NPs, where drugs encapsulated within NPs can escape multidrug resistance regulated by p-glycoprotein transporters [15]. Among NPs, biodegradable polymers of synthetic or natural origin are widely favored for drug loading and delivery, since they metabolize and require no surgical removal after drug release. Synthetic polymers are utilized in drug delivery due to their uniformity and ease of manufacture as well as scale-up. Unlike phospholipids, synthetic polymers are inert materials with no oxidative by-products released upon degradation and they possess versatile properties that can be manipulated to suit their intended use [16,17,18]. Several synthetic polymers have been shown to offer advantages in drug delivery, such as poly (lactic acid) (PLA), polycaprolactone (PCL), poly (butyl-cyanoacrylate) (PBCA), poly (glycolic acid) (PGA), poly (lactic-co-glycolic acid) (PLGA) and poly (amino acids) [19]. The choice of polymer depends on the final application of the NPs and their behavior can be affected by their crystalline or amorphous nature, porosity, biocompatibility and degradation profile. Depending on their chemistry, synthetic polymers tend to undergo swelling followed by erosion or degradation, with drug release taking place by both diffusion, during the early stages of release, and degradation, during the later stages of release [20].

Several techniques are used to manufacture drug-loaded polymeric NPs, such as solvent evaporation, nanoprecipitation, emulsification/solvent diffusion, salting out and supercritical fluid (SCF) technology [21]. Drugs can be formulated into NPs as either nanospheres or nanocapsules. Nanospheres are compact spheres where the drug is either dissolved or dispersed in a polymer matrix, with dissolved drugs forming homogeneous systems, while dispersed drugs tend to form heterogeneous systems. With nanocapsules, the drug is surrounded by a polymeric coat. Drugs can either be loaded onto pre-manufactured NPs by covalent or non-covalent interactions or loaded into the NPs during their manufacture. The efficiency of the drug loading is controlled by drug solubility, polymer molecular weight, functional groups and the type of interaction between the polymer and drug [22,23,24].

Hydrophilic drugs are difficult to formulate and encapsulate into the matrix of a hydrophobic polymer. This is because they have poor affinity towards hydrophobic polymers and they favor the water phase during the emulsification process, which results in poor encapsulation efficiency (EE). In addition, the large surface area of the NPs can lead to premature drug release due to a large amount of the drug being on the surface of NPs, instead of being encapsulated on the inside. PCL is a semi-crystalline and slow-degrading polymer, which can alleviate the issue of rapid drug release. However, there are no publications on manipulating PCL to increase its efficiency in encapsulating hydrophilic drugs. PCL degradation is slower than PLGA, which enhances its application in extending drug release for periods exceeding one year. Moreover, it has the advantages of high permeability to small drug molecules. A study [25] had previously reported sustained release of proteins using PCL. Other advantages of PCL that encourage its use over PLGA are: PCL is considered more economic than PLGA, it does not induce an acidic environment upon degradation, and it provides sustained release and biodegradation over longer periods of time, which would in turn have less impact on homeostasis [26].

Our preliminary studies demonstrate that the solvent evaporation technique is efficient in producing spherical and monodisperse PCL NPs with reasonable stability. Therefore, we selected this technique to develop PCL NPs with tailored mechanical properties to enable a high encapsulation efficiency of IRH. IRH is a potent hydrophilic chemotherapy that inhibits topoisomerase, leading to the inhibition of DNA replication [27]. The mechanism of action of IRH is not limited by MGMT methylation. Additionally, IRH and its highly potent metabolite (SN-38) are not substrates of the p-glycoprotein transporter and therefore will not face the challenge of MDR [28]. However, IRH can face difficulty crossing the BBB by passive diffusion because its molecular weight is 677 Da, which is above the limit (160 Da) for a hydrophilic drug. Moreover, IRH undergoes hydrolysis at physiological pH (7.4), which converts the active lactone form (stable below pH 5) to its less active carboxylate form (stable above pH 8) [29,30]. Therefore, it is necessary to protect the active form of the drug by shielding it from the physiological conditions, until reaching the acidic environment of the tumor. This study will investigate the influence of different parameters such as drug and polymer concentration; pH; type, molecular weight and concentration of surfactants; PCL amorphization and use of electrolytes on the size, polydispersity index (PDI), surface charge and encapsulation efficiency. The release profile and cytotoxicity will be evaluated for the optimized IRH-loaded PCL NPs.

## 2. Materials and Methods

### 2.1. Chemicals

PCL (14,000—50,000 g/mol), polyvinyl alcohol (PVA) (13,000–23,000; 89,000–98,000 g/mol), sucrose, olive oil, dioctyl-sulfo-succinate sodium, polyvinyl pyrrolidone (PVP) (40,000 g/mol), sodium chloride, paraffin oil, span-80, hydrochloric acid (HCL) and tween-20 were purchased from Sigma-Aldrich (Dorset, Gillingham, UK). IRH was purchased from LGM Pharma (Erlanger, KY, USA). Acetonitrile and dichloromethane (DCM) were purchased from ThermoFisher Scientific (Loughborough, UK).

### 2.2. IRH Solubility

An over-saturated solution was formed by dissolving 40 mg IRH in 1 mL of each of deionized water (dH_2_O) (pH 5.9) and PBS (pH 7.4). After 48 h, samples were centrifuged and the supernatant analyzed by high performance liquid chromatography (HPLC). Data were represented as mean (n = 3) ± standard deviation (SD).

### 2.3. IRH Stability

First, 1 mg of IRH was dissolved in 5 mL of dH_2_O and 10 mL of PBS and the solutions stored at 8 °C, 25 °C and 37 °C for 16 days. Samples were taken after 1, 2, 4, 8, 12 and 16 days and analyzed via HPLC. Data were represented as mean (n = 3) ± SD.

### 2.4. Solvent Evaporation Technique

PCL-IRH NPs were prepared using both the single and double emulsion solvent evaporation techniques by optimizing different parameters that influence the size, surface charge and EE of the formulated NPs.

#### 2.4.1. Single Emulsion (Oil in Water (O/W))

Briefly, the required amounts of PCL and IRH (Table 1) were dissolved in DCM, which was subsequently introduced dropwise into the aqueous phase containing varying concentrations (Table 1) of surfactant. The solution was then sonicated on an ice bath using a probe sonicator (ThermoFisher Scientific, Loughborough, UK) for various durations (Table 1), with solvent evaporation taking place for 4 h under gentle stirring at room temperature.

#### 2.4.2. Single Emulsion (Water in Oil (W/O))

First, 6 mg of IRH was dissolved in dH_2_O and emulsified via sonication in DCM containing 108 mg of dissolved PCL. Sonication was performed for 10 min on an ice bath and the solvent evaporated over 4 h at room temperature to produce SE15.

#### 2.4.3. Single Emulsion (Oil in Oil (O/O))

IRH-loaded NPs were produced using a modified single emulsion method (SE16). First, 108 mg of PCL and 6 mg of IRH were dissolved in 10 mL of DCM to form the first oil phase (O1). Then, 0.3% of dioctyl-sulfo-succinate sodium was dissolved in olive oil (O2). O1 was emulsified into 25 mL of O2 with sonication performed for 10 min at 50% amplitude on an ice bath. Solvent evaporation took place at room temperature over a period of 3 h. The formulation was subsequently centrifuged at 9500 for 20 min and washed twice using n-hexane. The pellet was collected by drying overnight at room temperature [31].

#### 2.4.4. Double Emulsion (Water in Oil in Oil (W/O/O))

A modified double emulsion method was investigated for the production of IRH-loaded NPs (DE1). First, 12 mg of IRH was dissolved in 0.5 mL of dH_2_O to form W1. Then, 108 mg of PCL was dissolved in 2 mL of DCM to form O1. W1 was added dropwise to O1 under gentle stirring. This first emulsion was subsequently added to 50 mL of liquid paraffin as the second organic phase (O2), which contained 0.3% span-80 as a surfactant. Sonication with 50% amplitude took place for 10 min on an ice bath. Then, the solvent was evaporated at room temperature over a period of 4 h. Centrifugation (Beckman Coulter Centrifuge, Buckinghamshire, UK) at 9500 rpm was used to collect the NPs, washing twice with n-hexane. The pellet was then collected by drying overnight at room temperature [32].

#### 2.4.5. Double Emulsion (Water in Oil in Water (W/O/W))

DE2 was prepared by dissolving the required amount of IRH in dH_2_O to form the aqueous phase (W1) and the required amount of PCL in DCM to form the organic phase (O). W1 was added dropwise to the organic phase, under gentle stirring, to form the first emulsion, which was subsequently added to the required volume of the external aqueous phase (W2) containing the required amount of PVA surfactant. Following the double emulsification process, sonication was performed on an ice bath, followed by solvent evaporation under gentle stirring at room temperature (Table 2).

##### The Influence of the Inclusion of Matrix Material in W/O/W

DE3 was prepared by dissolving 6 mg of IRH in 3 mL dH_2_O containing 2% sodium alginate to form the inner aqueous phase (W1). W1 was emulsified into 10 mL of DCM containing 108 mg PCL and 1% span 80 as a surfactant to form the W/O emulsion. This emulsion was further emulsified into the outer aqueous phase (W2) containing 100 mL of dH_2_O with 1.25% tween 80 [33].

##### The Influence of Using Amorphized PCL W/O/W

DE4 was prepared by using amorphous PCL and the same process parameters as DE2.

##### The Influence of the Addition of Electrolytes to the Aqueous Phase W/O/W

DE5 and DE6 were prepared using the double emulsion technique and the same process parameters as DE4. The first aqueous phase contained 10 mg/mL NaCl, while the second aqueous phase contained 2.5% NaCl [34].

### 2.5. The Influence of Different Parameters on Size, Charge and EE of Optimized NPs

Following the optimization of EE, other parameters were further investigated, as shown in Table 2, using 50% sonication amplitude for 10 min.

### 2.6. Collection and Lyophilization of NPs

NP formulations prepared by single emulsification were collected by centrifugation at 9500 rpm. Further optimization involved using a higher speed of 24,500 rpm to enhance the yield of the nanoparticles. The nanoparticles were then washed twice with dH_2_O to remove the free drug, frozen overnight at −80 °C in 5% sucrose solution and subsequently lyophilized at 0.01 mbar and −85 °C for 48 h. 

### 2.7. Morphology

The morphology of the IRN-loaded PCL NPs was analyzed by transmission electron microscopy (TEM) with a JEM 1010 electron microscope at a voltage of 80 80kV. The NPs were suspended in dH_2_O, deposited on a copper grid and dried before measurement.

### 2.8. Measurement of Particle Size and Zeta Potential

First, 2 mg of the lyophilized NPs was dispersed in 3 mL of dH_2_O and analyzed for their hydrodynamic diameter, PDI and zeta potential using a dynamic light scattering (DLS) Zetasizer (Malvern, Worcestershire, UK). Data were represented as mean (n = 3) ± SD.

### 2.9. Measurement of Encapsulation Efficiency

The supernatant for the different NP formulations was analyzed by HPLC to determine the un-encapsulated drug (free drug). Encapsulation efficiency was calculated using the following equation:

$$\mathrm{EE}\;(\%)=\frac{\mathrm{Initial}\;\mathrm{drug}\;\mathrm{amount}-\mathrm{Drug}\;\mathrm{in}\;\mathrm{the}\;\mathrm{supernatant}}{\mathrm{Initial}\;\mathrm{drug}\;\mathrm{amount}}\;\times \;100$$

### 2.10. Release Study

First, 25 mg of the IRH-loaded NPs was dispersed in 25 mL of PBS release media and stirred at 200 rpm at 37 °C. Then, 0.5 mL samples were removed at 0, 1, 3, 5, 7, 9, 14, 16 days and replaced with fresh media. The samples were centrifuged and the supernatant analyzed for their IRH content using HPLC. The percentage of drug release was determined as mean (n = 3) ± SD using the following equation:

$$\mathrm{Drug}\;\mathrm{release}\;(\%)=\frac{\mathrm{Released}\;\mathrm{IRH}}{\mathrm{Total}\;\mathrm{IRH}}\times 100\;$$

### 2.11. IRH HPLC Methodology

IRH was analyzed using Agilent HPLC (Agilent Technologies 1260 infinity II) with a quaternary gradient pump. The separation was performed using a C18 column (150 mm × 4.6 cm), particle size 5 μm (ThermoFisher Scientific, Loughborough, UK), at 25 °C. The injection volume was 20 μL, with a run time of 10 min and a flow rate of 1.00 mL/min. The mobile phase was composed of ion pair solution (solution A): 1.2 g octane-l-sulfonic acid in 500 mL dH_2_O and solution B: 13.6 g potassium dihydrogen phosphate dissolved in 500 mL dH_2_O. Solution A, solution B and acetonitrile were in the ratio of 30:30:40 *v*/*v*/*v*. The pH was adjusted to 3 using orthophosphoric acid. The detector UV wavelength was set at 265 nm.

### 2.12. In Vitro Cell Culture and Cytotoxicity Study

Unfixed tumor core tissue was collected directly from recurrent glioblastoma patients undergoing craniotomies at the Queen Elizabeth Hospital, in accordance with ethical approval (application number: 19-337) from the Human Biomaterials Resource Centre (HBRC). The samples were immediately placed in collection fluid and transported to the laboratory. Extraction of the tumor cells from the tissue and culturing of the subsequent cells were performed using standard methods. Cells were maintained using Dulbecco’s modified Eagle’s medium/nutrient mixture F-12 ham supplemented with 10% FBS (foetal bovine serum), penicillin/streptomycin, sodium pyruvate (Sigma-Aldrich, Dorset, Gillingham, UK) and minimum essential medium non-essential amino acid solution (MEM NEAA) (ThermoFisher Scientific, Loughborough, UK). The cells were plated onto 96-well flat-bottomed microtiter plates at a density of 10,000 cells per well and cultured in the presence of free IRH (low, medium and high concentrations equivalent to 1, 10 and 100 μM, respectively). The NP formulations were selected in low, medium and high concentrations equivalent to 1, 10 and 100 μM of encapsulated drug, respectively. Cytotoxicity testing was performed using the standard 3-(4,5-Dimethylthiazol-2-yl)-2,5-Diphenyltetrazolium Bromide (MTT) assay after 1, 3, 5, 7, 9 and 11 days of treatment, with all experiments performed in triplicate (n = 3) ± SD. MTT was purchased from Sigma-Aldrich (Dorset, Gillingham, UK). 

### 2.13. Statistical Analysis

The statistical analysis was carried out with GraphPad Prism, version 9. Two-way ANOVA (Analysis of variance) was used for multiple comparisons. The statistical significance was presented as: *p* ≤ 0.0001, *p* ≤ 0.05. Non-significance was presented for *p* > 0.05. Data were presented as mean (n = 3) ± SD.

## 3. Results

### 3.1. IRH Solubility and Stability

**Figure 1:**
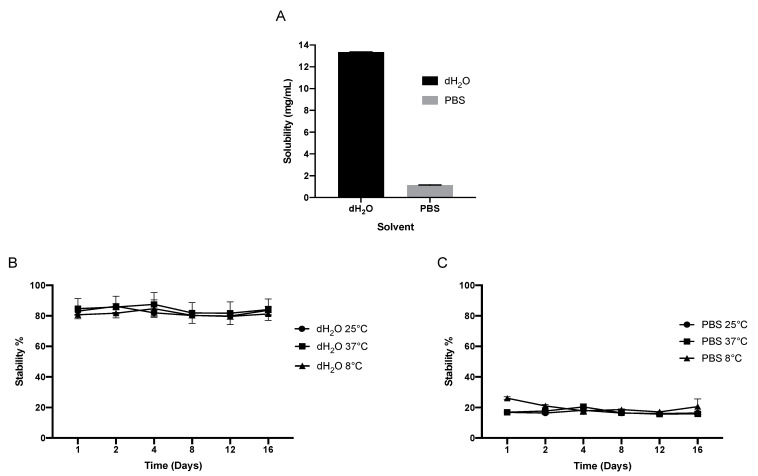
Solubility (**A**) and stability of IRH in both dH_2_0 (**B**) and PBS (**C**) over 16 days at 8, 25 and 37 °C.

Figure 1A demonstrates that IRH is more soluble in dH_2_O compared to PBS. IRH has a solubility of 13.34 ± 0.046 mg/mL and 1.15 ± 0.056 mg/mL in dH_2_O (pH 5.9) and PBS (pH 7.4), respectively, which correlates with the literature [35]. In water, IRH retains greater than 80% of its stability over 16 days, with temperature having no effect (Figure 1B). However, in PBS, IRH loses more than 70% of its stability within 24 h, which remains constant over 16 days, with temperature having no effect (Figure 1C).

### 3.2. Single Emulsion Optimization Parameters

The aim of this study was to produce NPs with an average hydrodynamic diameter less than 500 nm, low PDI, an EE greater than 50% and high stability, which was based on their zeta potential. Small NPs are characterized by a large surface area, which could enhance drug dissolution and release [36].

#### 3.2.1. Parameters Influencing the Size, PDI and Surface Charge

**Figure 2:**
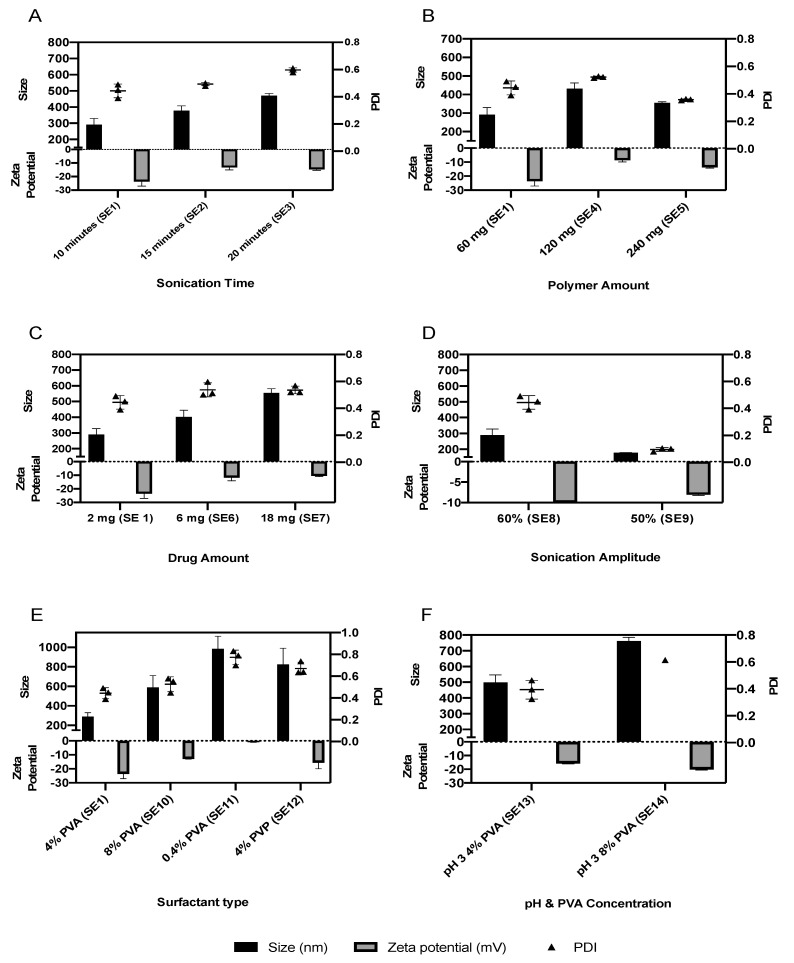
The impact of sonication time (**A**), polymer amount (**B**), drug amount (**C**), sonication amplitude (**D**), surfactant type (**E**), pH and PVA concentration (**F**) on the average particle size, PDI and zeta potential of the nanoparticles manufactured using the single emulsion technique.

Size optimization using the single emulsion technique was started by investigating three different durations of sonication (10, 15 and 20 min), as shown in Figure 2A. A duration of 10 min provided the most optimum results of monodisperse NPs (PDI < 0.5), with the smallest average hydrodynamic diameter of 290.90 ± 38.49 nm and a zeta potential of −23.7 ± 3.3 mV. The formulation parameters were then investigated for their impact on size and surface charge. Increasing the amount of PCL from 60 to 120 mg increased the particle size and PDI of the NPs, while a further increase in the amount of PCL reduced the particle size and PDI of the NPs (Figure 2B). Additionally, increasing drug loading from 2 to 18 mg resulted in an increase in particle size up to 554.3 ± 27.2 nm, with a zeta potential of −10.6 ± 0.5 mV (Figure 2C). Similarly, increasing the PVA concentration from 4 to 8% increased the size and PDI of the NPs (Figure 2F). PVP was investigated as a stabilizer [37]; however, it resulted in an increase in both size and PDI (Figure 2E). Additionally, a low PVA concentration of 0.4% increased the size of the NPs (Figure 2E).

Further optimization was performed by investigating the influence of sonication power on the size of the NPs. By switching from 60 to 50% sonication amplitude, the average hydrodynamic diameter of the NPs was reduced from 290.9 ± 38.5 nm to 178.6 ± 0.7 nm. Therefore, an amplitude of 50% was considered as the optimum sonication power for preparing the NP formulations (SE9), which was considered for further analysis by HPLC to determine the EE.

#### 3.2.2. Parameters Influencing EE

Following size optimization, the drug content of SE9 was analyzed by HPLC and was very low (7.5%), which led us to investigate further parameters that could enhance the EE. By switching the emulsification process to water in oil as in SE15, the formulation stability was compromised, leading to the formation of irreversible aggregates.

Since IRH is a hydrophilic drug and tends to escape to the water phase, which limits its encapsulation, a method devoid of water was selected (O/O) to overcome this problem. SE16 was prepared using the O/O method, which was previously proven successful in encapsulating a water-soluble drug in PCL microparticles [38]. This method enhanced the EE to 40.5% when the parameters were adjusted in line with our optimized formulation parameters (SE9); however, the size was not in the nanometer range and the particles had poor stability, represented in the form of aggregates precipitating out of the emulsion. As a result, it was decided to focus on improving the EE of the O/W emulsion via lowering the pH of the aqueous phase and the addition of NaCl to the aqueous phase; however, both strategies resulted in no drug encapsulation. Finally, the PCL polymer was converted to the amorphous form by heating to its melting point and subsequently quench cooling on dry ice until solidification [39,40]. Using the amorphous PCL increased the EE to 12.1% (SE17), which was still considered low; however, it demonstrated that the amorphous PCL had a higher EE than the crystalline PCL. Based on the above observations, it has been concluded that the single emulsion technique is not suitable for the manufacture of IRH-loaded PCL NPs with an acceptable EE above 50%.

### 3.3. Double Emulsion Optimization Parameters

**Figure 3:**
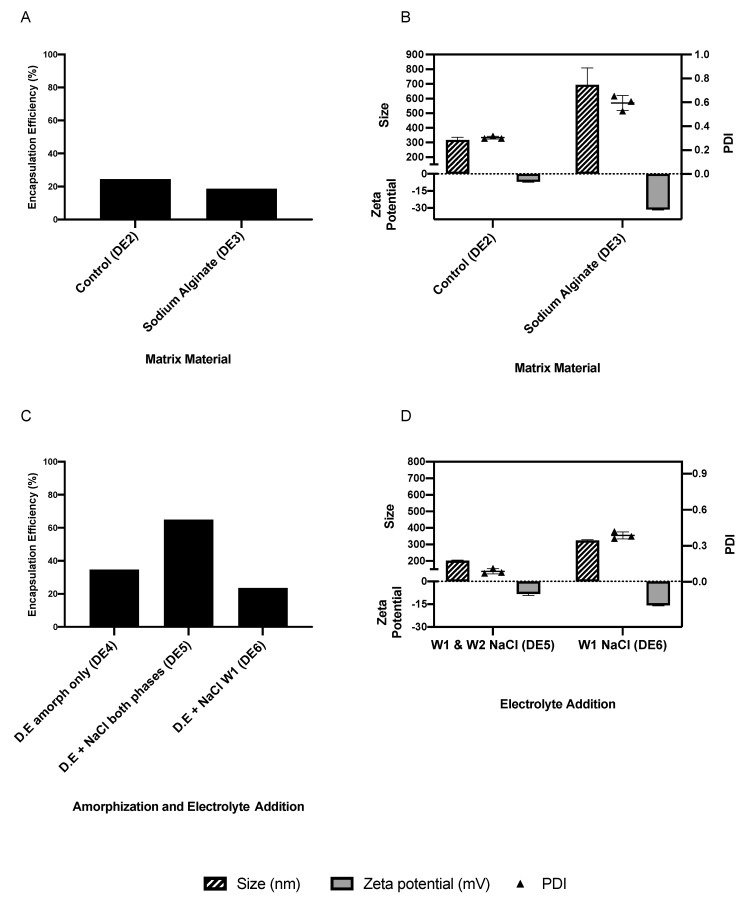
The impact of matrix material, PCL amorphization and electrolyte addition on the average particle size, PDI, zeta potential (**A**,**C**) and EE of nanoparticles (**B**,**D**) manufactured using the double emulsion technique.

**Figure 4:**
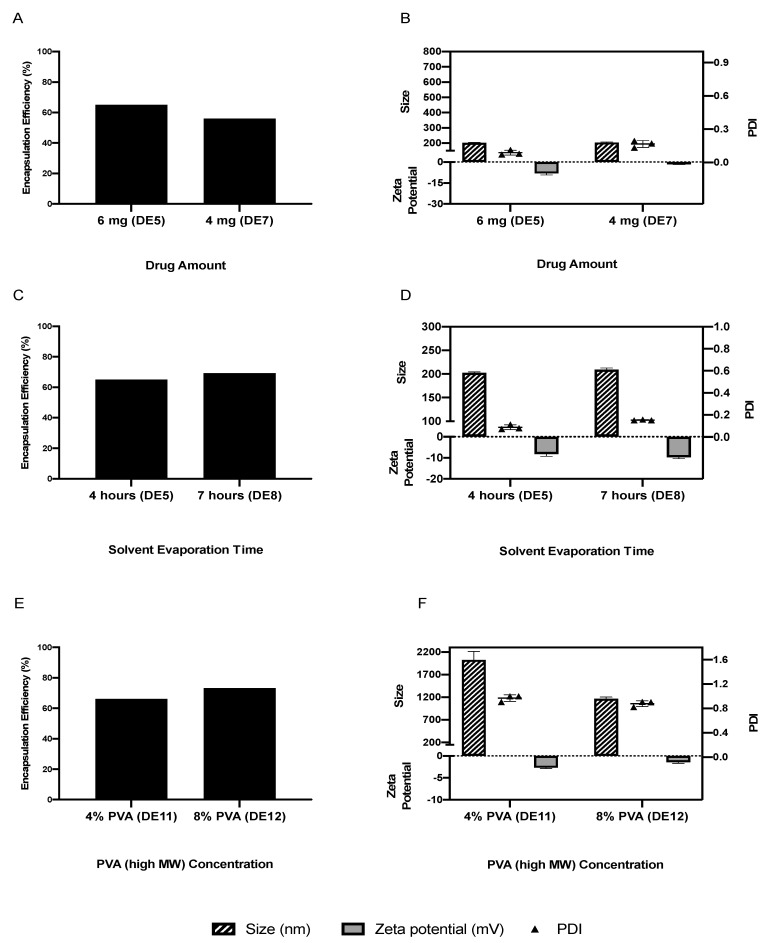
The impact of drug amount (**A**,**B**), solvent evaporation time (**C**,**D**) and high-MW PVA (**E**,**F**) on the average particle size, PDI, zeta potential and EE of optimized nanoparticles manufactured using the double emulsion technique.

**Figure 5:**
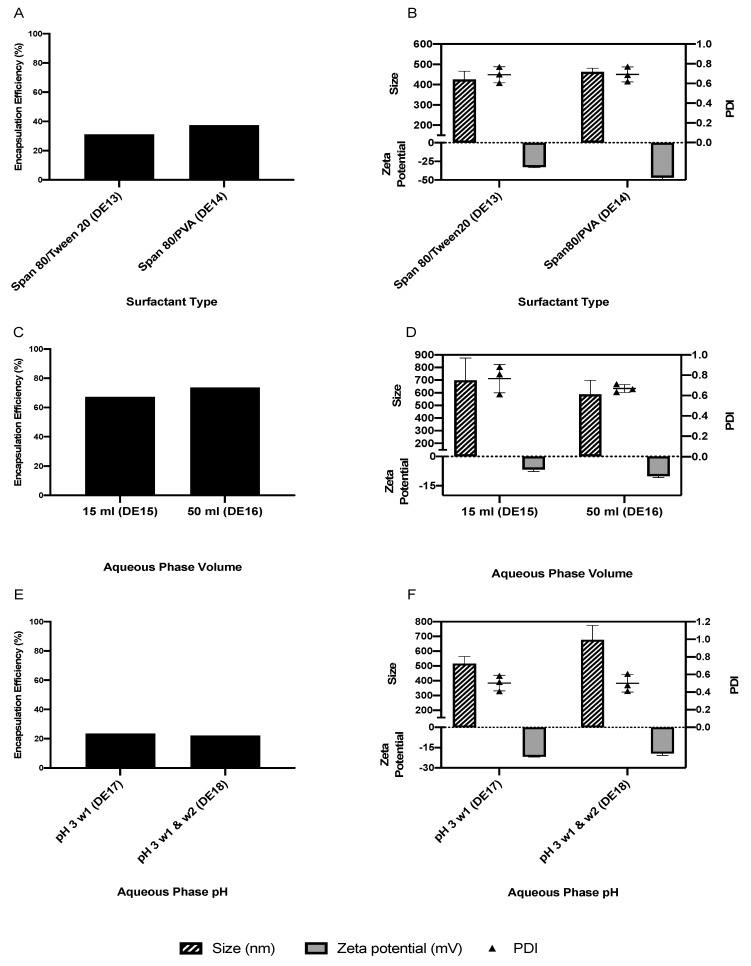
The impact of surfactant type (**A**,**B**), aqueous phase volume (**C**,**D**) and aqueous phase pH (**E**,**F**) on the average particle size, PDI, zeta potential and EE of optimized nanoparticles manufactured using the double emulsion technique.

DE1 was prepared using the W/O/O emulsification technique, which produced an EE of 11.1%, but the size of the particles exceeded the nanometer range and thus it was excluded from the study. By using the W/O/W double emulsion technique, it was possible to achieve EEs greater than the acceptable 50%, proving that the double emulsification technique was more suited to the manufacture of IRH-loaded PCL NPs than the single emulsification technique, which is due to the hydrophilic nature of IRH. The W/O/W double emulsification produced NPs with EEs ranging from 18.7 to 73.7% depending on the process and formulation parameters (Figure 3, Figure 4 and Figure 5).

Attempts to improve the EE started with the inclusion of a matrix material (Na alginate) to provide more matrix to entrap the drug. This approach (DE3) provided an EE of 18.7% (Figure 3A), which was considered too low, while increasing the particle size (Figure 3B). Based on the positive EE data for the amorphous PCL using the single emulsion technique, it was investigated using the double emulsion technique. The use of amorphous PCL (DE4) increased the EE to 34.8% (Figure 3C), while the addition of NaCl to both aqueous phases in combination with the amorphous PCL (DE5) increased the EE to 65.0% (Figure 3C), which was higher than the use of amorphous PCL on its own or the addition of NaCl to W1 (DE6). Furthermore, neither approach increased the size of the NPs to above 324 nm (Figure 3D).

After achieving an acceptable EE (65.0%), several other parameters were investigated for their influence on EE, size and charge (Figure 4 and Figure 5). Reducing the amount of IRH from 6 to 4 mg decreased the EE to 56.0%, with limited impact on the size (Figure 4A,B). Increasing the solvent evaporation time from 4 to 7 h improved the EE slightly from 65.0 to 69.2% (Figure 4C), with a slight increase in particle size and zeta potential (Figure 4D). A duration greater than 7 h (DE9 and 10) resulted in the formation of aggregated particles not in the nanometer range. Therefore, an evaporation time of 4 h was selected as the optimum time for providing a suitable size, charge and EE. Using a PVA with a high average molecular weight (89,000 g/mol) (DE11 and 12) increased the EE to 73.2% (Figure 4E); however, the particle size was outside the nanometer range, with a high PDI (Figure 4F), and thus it was excluded from further study. The use of two surfactants with different hydrophilic–lipophilic balance (HLB) values (DE13 and 14) reduced the EE to 31.2 and 37.5% (Figure 5A) while increasing the size, PDI and zeta potential of the NPs (Figure 5B). Increasing or decreasing the volume of the aqueous phase to above or below 25 mL resulted in an increase in EE (Figure 5C); however, both particle size and PDI were also increased (Figure 5D) and thus 25 mL was selected as the optimum volume for the W2 aqueous phase. Decreasing the pH of either one or both of the aqueous phases reduced the EE (Figure 5E) while also increasing particle size, PDI and surface charge (Figure 5F). A summary of the EE% achieved using different methods and parameters is shown in Table 3.

Based on the optimization data above, it was decided that the double emulsion technique, using amorphous PCL with the addition of NaCl to both aqueous phases (DE5), was the optimum condition for the manufacture of IRH-loaded PCL NPs. The DE5 NPs were selected for further characterization of their morphology, drug release and cytotoxicity.

### 3.4. Characterization of the Optimized IRH-Loaded PCL Nanoparticles

**Figure 6:**
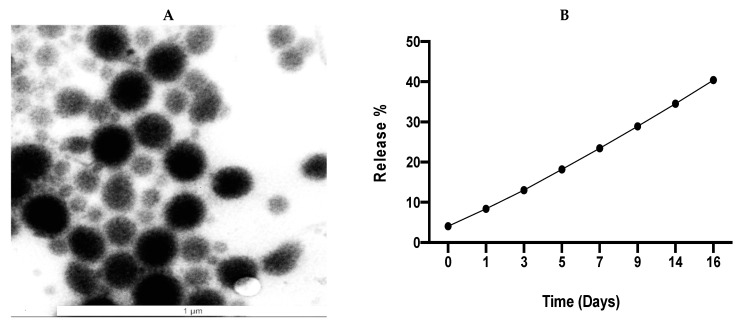
TEM image of DE5 showing average particle size of 149.2 ± 13.5 nm for 14 particles (**A**) and in vitro cumulative release of the IRH-loaded PCL nanoparticles (**B**).

A TEM image of the optimized IRH-loaded PCL NPs (DE5) is presented in Figure 6A. It demonstrates that the nanoparticles are a group of uniform, spherical particles with smooth edges that are within the nanometer range, with an average particle size of 149.2 ± 13.5 nm, which is lower than the 202.1 ± 2.1 nm determined by DLS. This is due to DLS measuring the hydrodynamic diameter, which is the size of the NPs plus the liquid layer around them, while TEM is performed on dried NPs and gives the actual size of the NPs. However, TEM is an image of a much smaller sample size of the NPs compared to DLS. The TEM image suggests that the NPs are polydisperse, which does not agree with the PDI value of the DLS. However, the sample preparation for TEM can lead to the NPs forming layers on top of each other, which makes them resemble one large particle, giving the impression of a polydisperse system [41]. The in vitro cumulative release demonstrates that the nanoparticles provide zero-order sustained release of IRH, releasing 40.4 ± 1.5% of their IRH content over the 16 days (Figure 6B). Table 4 shows the kinetic parameters for the zero-order, Higuchi and Korsmeyer–Peppas release models. The coefficient of determination (R^2^) values demonstrate that the release fits all three models, with R^2^ values of 0.9991, 0.9595 and 0.9958, respectively. This demonstrates that their release is zero-order and diffusion-controlled, while a diffusional exponent (n) value of 0.6894 for the Korsmeyer–Peppas model suggests non-Fickian, anomalous diffusion, which is to be expected for NPs due to their small size and short diffusion pathway. Based on the diffusional exponents and release kinetic constants listed in Table 4, the mechanism of release is non-Fickian diffusion, with the IRH released via a combination of diffusion through the PCL and swelling of the PCL.

**Figure 7:**
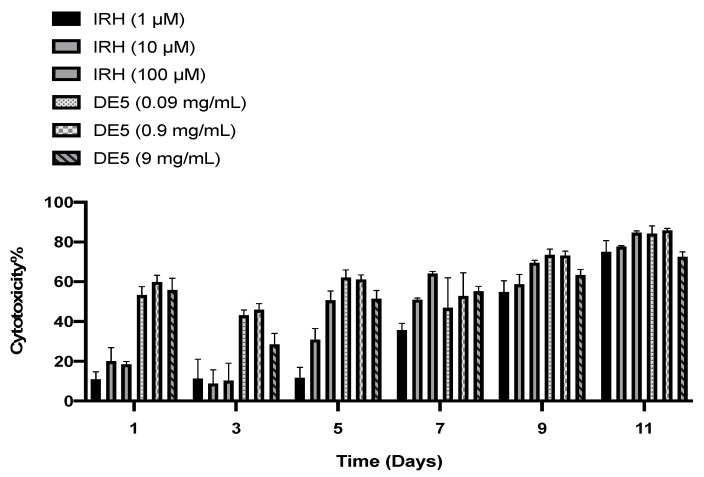
The cytotoxicity of free IRH and DE5 formulation on primary HGG cells.

The cytotoxicity of the nanoparticles was compared to the unformulated IRH at three different doses over 11 days (Figure 7). The NPs were more cytotoxic than the unformulated IRH across all doses, with the highest cytotoxicity achieved at day 11. However, by day 5, there was no significant difference between the cytotoxicity of the high-dose unformulated IRH and the high-dose IRH-loaded PCL NPs, while, by day 7, there was no significant difference across all doses up to day 11.

## 4. Discussion

PCL NPs have great potential for drug delivery due to their slower biodegradation when compared to PLGA, which allows them to deliver drugs into cancerous tissue over a longer period of time, thus reducing the dosing interval. However, it is notoriously difficult to encapsulate drugs into PCL NPs due to its high crystallinity and the fact that drug entrapment occurs in the amorphous regions [42]. This was reported in a previous study where the percent EE of 5-fluorouracil in PCL NPs ranged from 4.3 ± 0.4 to 35.2 ± 3.1 [43]. Another study reported low drug release of 5% over a period of one week from PCL NPs [44]. These challenges have led to limited publications on PCL NPs when compared to PLGA NPs. This paper aimed to determine the most appropriate emulsification technique and formulation parameters to manufacture IRH-loaded PCL NPs with an acceptable EE, size and zeta potential. The optimum NPs were subsequently evaluated for their morphology, IRH release and cytotoxicity.

Both single and double emulsion techniques were evaluated using different process and formulation parameters to identify the optimum conditions for encapsulating the water-soluble IRH drug into PCL NPs.

### 4.1. Parameters Influencing Size, PDI and Surface Charge of Nanoparticle Formulations

#### 4.1.1. Sonication Time and Amplitude

In Figure 2A, increasing the sonication time from 10 to 20 min resulted in an increase in the size of the NPs from 290.9 ± 38.5 nm to 471.1 ± 12.7 nm. This is due to overheating of the solution. resulting in agglomeration, which has been previously reported [45]. Sonicator probes induce the formation of vapor cavities in the solution by the effect of mechanical vibration: this process is termed cavitation [46]. This temporary acoustic cavitation can enhance emulsification. Cavitation can take place over two stages: 1. The low-frequency acoustic waves lead to an unstable oil–water interface; 2. Temporary cavitation bubbles develop. The cavitation threshold is the minimum amount of energy required to generate cavitation. In order for the emulsification process to take place, the sonication power should be higher than the cavitation threshold [47]. In our study, a sonication amplitude of 50% allowed for emulsification to take place and produce smaller NPs compared to a higher sonication power. Previous studies had reported similar observations, where ultrasonication was effective in separating the agglomerated NPs, but further ultrasonication led to re-agglomeration of the NPs [48]. In another study, elevated levels of ultrasonication resulted in agglomerated NPs [49]. This highlights that when the heating caused by sonication dominates over the cavitation power, it reduces the viscosity of the suspension and increases the thermal motion of the particles. This allows a higher collision rate between the accelerated particles, which results in increased particle interaction and agglomeration [48]. Agglomeration can also take place via the adhesion of particles if van der Waals attractive forces are predominant over the repulsive forces [50].

#### 4.1.2. Drug and Polymer Amount

Increasing the amount of IRH from 2 to 18 mg using the single emulsion technique increased the size of the NPs from 290.9 ± 38.5 nm to 554.3 ± 27.2 nm. This is due to more drug particles accumulating on the surface of the polymeric NPs, as well as the low stability of the solution, leading to agglomeration of the NPs. Similar studies reported an increase in the particle size upon increasing the amount of drug [51,52]. Other research has suggested that increased phase viscosity occurs when the amount of drug is increased, leading to a larger particle size [53]. Meanwhile, Chorny et al. (2002) reported no significant impact on particle size when the amount of drug was increased [54]. Increasing the amount of PCL to 240 mg reduced the particle size, which suggests that this polymer amount was sufficient to entrap more drug particles, preventing them from adsorbing on the surfaces of the nanoparticles and increasing their diameter.

#### 4.1.3. Surfactant Concentration

Surfactants play an important role in reducing the surface tension between water and oil if used at the correct concentration. Low concentrations of surfactants do not reduce the surface tension as there is not enough surfactant to cover the interfacial surface between the organic and aqueous phases and thus are unable to stabilize the nanodroplets within the emulsion and prevent them from agglomerating [36]. The results demonstrate that 0.4% PVA (SE11) resulted in a large particle size (984.6 ± 129.6 nm) and a polydisperse formulation (PDI > 0.5), which is in accordance with a previous study that used 0.5% PVA for stabilizing PLGA NPs [36]. Furthermore, increasing the PVA concentration resulted in a decrease in the average size of the NPs produced by single emulsion (Figure 2E,F), which is in accordance with previous studies [55,56]. However, others have reported an increase in the particle size upon using high concentrations of PVA [57,58]. This discrepancy could be attributed to two different effects at high PVA concentrations: (1) increased stability between phases induced by the surfactant leads to a reduction in size; (2) increased viscosity of the aqueous phase results in poor mixing quality, thereby increasing size. The emulsification process and parameters used to produce the NPs would influence which of the above effects dominates over the other [59].

#### 4.1.4. Surfactant Type and Molecular Weight

Surfactants can impact particle size and are responsible for the overall formulation stability. PVA, when used as a single aqueous phase surfactant, produced smaller NPs with better stability, when compared to the PVP used in the study. Another study obtained large particle sizes when using PVA [60]. In our study, the PVA with high molecular weight (89,000 g/mol) produced particles of a size outside the nanometer range due to its high viscosity. Other groups made similar observations, which demonstrate that too much emulsifier can cause particle aggregation as it adsorbs onto the surface of the NPs [52].

### 4.2. Parameters Influencing Encapsulation Efficiency

#### 4.2.1. Single Emulsion Solvent Evaporation Technique

The W/O single emulsion solvent evaporation technique produced unstable particles that contained aggregates. The O/W single emulsion solvent evaporation technique produced stable NPs within the nanometer range; however, the amount of IRH encapsulated was low, with a maximum of 12.1% achieved when amorphous PCL was used. The O/O single emulsion solvent evaporation technique produced particles outside the nanometer range, with an EE of 40.5%. Emulsions prepared using oil as the continuous phase provide better encapsulation for hydrophilic drugs by reducing the drug from partitioning into the external continuous phase [31]. A comparative study was conducted using both O/O and O/W solvent evaporation techniques to encapsulate the hydrophilic drug Rifampicin in PCL microspheres. Their results provided EEs of 43.5 to 61.9% and 12.3%, respectively [38]. The only disadvantage of using this method for the IRH-loaded PCL NPs is that it does not produce particles within the nanometer range but could be suitable for producing microspheres.

The single emulsification technique has a poor record of encapsulation of hydrophilic drugs [61]. Previous studies highlighted that the hydrophilic drugs such as caffeine, salicylic acid and theophylline could not be entrapped within polylactic acid using the O/W single emulsification technique [62]. Therefore, we investigated the double emulsification technique with different formulation parameters to identify those that produce optimum IRH-loaded PCL NPs in relation to EE, size, PDI and zeta potential.

#### 4.2.2. Double Emulsion Solvent Evaporation Technique

The W/O/O double emulsion solvent evaporation technique has previously been used to increase the EE of a hydrophilic protein in PLGA microparticles [32]. However, our results demonstrate that this technique achieved an EE of 11.1%, with a particle size outside the nanometer range, which suggests that the additional oil phase increases particle size and reduces drug entrapment. It is theorized that using a W/O/W double emulsion solvent evaporation technique leads to the formation of a semipermeable membrane, which permits water to migrate through the organic phase. This in turn leads the internal droplets to either swell or shrink, based on the direction of the osmotic gradient [63]. Using this technique produced IRH-loaded PCL NPs with an acceptable particle size (less than 500 nm) and EE (greater than 50%); however, the parameters that reduce drug leakage into the aqueous phase had to be adjusted.

##### Matrix Material

The hydrophilic nature of IRH means that it readily moves into the aqueous phase, lowering its entrapment within the polymer matrix. Several studies on the emulsification of water-soluble drugs have reported low entrapment efficiency [64,65,66,67,68], while other studies have suggested that the use of a polymer matrix material, such as sodium alginate, can reduce the diffusion of the hydrophilic drug into the aqueous phase, increasing EE [69,70,71]. The use of sodium alginate as a matrix material (DE3) produced IRH-loaded PCL NPs with an EE of only 18.7% and resulted in large particle size (>500 nm) and was thus discontinued. The large particle size could be due to the gelation of the sodium alginate, leading to high viscosity and incomplete emulsification. Additionally, the amount of sodium alginate could be insufficient to entrap the drug, as reported elsewhere [72].

##### The Use of Amorphous PCL and the Addition of Electrolytes

PCL with a crystallinity of up to 70% reduces the EE of drugs that generally favor more amorphous cores [42]. Semi-crystalline polymers such as PCL have a higher proportion of hydrophobic blocks, which increases core crystallinity and reduces their drug encapsulation capacity due to drugs favoring the amorphous blocks of the polymer [73]. Using the double emulsion technique and amorphized PCL, IRH-loaded PCL NPs (DE4) with an EE of 34.8% were produced, which was enhanced to 65.0% with the addition of NaCl to both aqueous phases (DE5). However, the addition of NaCl to only aqueous phase W1 (DE6) resulted in a reduction in EE. Salts can enhance EE by producing an osmotic gradient and by forming porous particles. However, too many pores can lead to drug leakage. Similar to our results, the addition of NaCl with certain concentrations in both aqueous phases had previously improved the EE for protein-loaded microspheres [34]. By increasing the NaCl concentration in the internal phase W1, it forces the water to migrate as a result of the osmotic pressure gradient from the outer to the inner phase, causing swelling of the inner-phase droplets and the particles within. This swelling phenomenon causes breakage of the polymeric layer and release of the inner phase into the outer aqueous phase, which removes the drug during the encapsulation process. Therefore, at high salt concentrations, there is a possibility of damaging the double emulsion layer induced by the coalescence of the internal water droplets. On the other hand, the addition of NaCl to the external water phase could switch this phenomenon favorably. However, high salt concentrations in the external aqueous phase, regardless of the concentration of the salt in the W1 phase, could cause the water droplets to remain small in size, thereby keeping the porosity to a minimum. The results agree with these findings, with an improvement in the EE upon the addition of NaCl to both the internal (10 mg/mL) and external (2.5%) phases.

##### Amount of IRH

The reduction of the amount of IRH to 4 mg (DE7) reduced the EE of the NPs without much impact on the size. However, some studies suggest that the amount of drug does not play an important role in the EE as long as the polymer amount remains fixed and that the internal morphology of the particles is not affected by the drug loading [36,53]. Due to IRH being a hydrophilic drug, reducing the amount of IRH in the aqueous phase reduces its partitioning into the oil phase, which reduces the EE. Increasing the amount of IRH in the aqueous phase increases its partitioning into the oil phase, increasing EE [74].

##### Solvent Evaporation Time

Increasing the solvent evaporation time from 4 (DE5) to 7 (DE8) hours had a slightly positive impact on EE, increasing it from 65.0 to 69.2%; however, there was also a small increase in particle size from 202.1 ± 2.1 to 209.2 ± 3.3 nm, respectively. A duration longer than 7 h (DE9 and 10) reduced the stability of the formulations, leading to the formation of aggregates, with no NPs detected. The gradual decrease in the emulsion volume due to evaporation leads to an increase in viscosity, which impacts the electrostatic charge and equilibrium of the emulsion and leads to agglomeration during solvent removal [36].

##### Surfactant Concentration

High concentrations of surfactants can increase the viscosity of the emulsion, leading to decreased stability, especially due to the gelatinization of PVA as a result of the formation of hydrogen bonds with the OH groups in the PVA during the formation of the NPs. Such a highly viscous emulsion could prevent the sonication energy from reaching all the droplets to shatter them into NPs. Moreover, the drug content of such viscous emulsions is usually high since the drug molecules have limited diffusion into the aqueous phase. Such opposing effects of the surfactant depend on the properties and concentration of the surfactant, as well as the formulation parameters, which will dictate which effect will dominate [36]. Maaz et al. improved the EE of the hydrophilic drug Gatifloxacin by increasing the surfactant concentration in the formulation [75]. It is theorized that increasing the surfactant concentration decreases the partitioning of a hydrophilic drug into the aqueous phase, thus enhancing its EE. Increasing the concentration from 4% (DE19) to 8% (DE20) for both the low- and high-MW PVA (DE11 and DE12) produced particles with EEs of 29.0, 43.3, 66.1 and 73.2%, respectively. Despite the high-MW PVA resulting in EEs above 50%, this formulation was abandoned due to the size of the particles being outside the nanometer range.

##### Surfactants with Different HLB Values

The oil layer of a W/O/W double emulsion sits between both aqueous phases and thus requires a minimum of two surfactants with different HLB values in order to form a stable emulsion. A surfactant with a low HLB value is required to emulsify the water in the oil (W/O) component, while a surfactant with a higher HLB value is required to further emulsify the W/O component into the water phase to form the W/O/W [61]. Using tween-20 as the aqueous phase surfactant and span-80 as the organic phase surfactant (DE13) produced IRH-loaded PCL NPs with an EE of 31.2%, while using PVA as the aqueous phase surfactant and span-80 as an organic phase surfactant (DE14) achieved an EE of 37.5%. Although the NPs produced using this approach had an optimum size and high stability in terms of zeta potential, they did not achieve the optimum EE of greater than 50% and were therefore not investigated further.

##### Aqueous Phase Volume

Increasing or decreasing the volume of the aqueous phase to above or below 25 mL resulted in an increase in EE (Figure 5C) as well as particle size and PDI (Figure 5D). In addition, 15 and 50 mL of aqueous phase resulted in a particle size of 698.9 ± 175.8 and 587.1 ± 111.3 nm, respectively. This change in the ratio of aqueous and organic phases negatively impacts the emulsification shear force required for the formation of the NPs. By increasing the volume, the shear force of sonication will be distributed over the large volume and will not be sufficient to break down all the particles to convert them to NPs. Likewise, a low aqueous phase volume will cause extra shear force and overheating of the small volume, leading to aggregation and a larger particle size [36]. Based on these results, 25 mL was confirmed as the optimum volume for the aqueous phase.

##### pH of the Aqueous Phase

Mohammady et al. found that by lowering the pH of the aqueous phase, the EE of IRH in PLGA NPs could be improved to above 50% [52]. However, reducing the pH of only the inner aqueous phase (DE17) or both the inner and outer phases (DE18) to a pH of 3 produced IRH-loaded PCL NPs with EEs of only 23.6 and 22.3%, respectively; therefore, this approach was not investigated any further.

### 4.3. Characterization of the Optimized IRH-Loaded PCL Nanoparticles

IRH-loaded PCL NPs with an acceptable EE (>50%), size (<500 nm) and charge were produced using the double emulsion technique, 6 mg of IRH, amorphous PCL and the addition of NaCl to both aqueous phases (DE5). These NPs were subsequently characterized for their morphology, drug release and cytotoxicity against primary HGG cells.

#### 4.3.1. Morphology and In Vitro Drug Release

TEM analysis demonstrated that the optimized IRH-loaded PCL NPs are a group of uniformly spherical particles with smooth edges that are within the nanometer range, with an average particle size of 149.2 ± 13.5 nm, which is lower than the 202.1 ± 2.1 nm determined by DLS. The in vitro drug release demonstrates a small burst on day 1 followed by slow and sustained release over a period of 16 days, with the NPs releasing 40.4 ± 1.5% of their IRH content. The small burst on day 1 is due to the optimization process, which has resulted in more IRH being encapsulated in the NPs and less on the surface. The hydrophobic nature of the PCL delays the penetration of water into the matrix core, which means that IRH release is controlled solely by diffusion, with no polymer erosion. The small size of the nanoparticles results in a negligible diffusion pathway, resulting in the NPs having a zero-order release profile.

To better understand the drug release kinetics, the data were fitted to zero-order (cumulative percent of drug release versus time), Higuchi (cumulative percent of drug release versus square root of time) and Korsmeyer–Peppas release models (log cumulative percent of drug release versus ln time) (Table 4). The release data fitted all three models, as represented by the high regression coefficients (R^2^) of 0.9991, 0.9595 and 0.9958, respectively. The correlation of the data to both the zero-order and Higuchi release models confirms that the release of the IRH is both zero-order and diffusion controlled. Drug release from polymeric NPs using the Korsmeyer–Peppas model can be described using the following equation: Mt/M^∞^ = kt*^n^* [74]. (M_t_/M^∞^) is the fraction of drug released at time t, k is the kinetic constant, and n is the exponent that correlates to a particular diffusion mechanism. Our results provided an n value of 0.6894, which corresponds to anomalous transport, which is non-Fickian diffusion. This is to be expected for NPs due to their small size and short diffusion pathway, while the inclusion of NaCl may have produced porous particles, which have been shown to have anomalous transport [76]. In non-Fickian anomalous diffusion, tr ≈ td, where tr is the polymer relaxation time and td is the solvent penetration time [77]. The release exponent n and K values were calculated from the slope of the straight line for the plot of log cumulative percentage of drug released versus ln time.

#### 4.3.2. Cytotoxicity

The initial cytotoxicity of the IRH-loaded NPs was significantly (*p* < 0.0001) higher than the unformulated IRH, which is due to the NPs penetrating the tumor cells faster than the free drug. Furthermore, nanoparticles are known to induce cytotoxicity by inducing apoptosis, depletion of ATP and necrosis [76]. This may also be increasing their cytotoxicity at day 1 and day 3. By day 5, there was no significant difference in cytotoxicity between the high dose of the NPs and the high dose of the free IRH, and by day 7, there was no significant difference in cytotoxicity between all doses of the NPs and all doses of the free IRH. This is due to the IRH having now penetrated and diffused into the HGG cells. Additionally, the IRH-loaded NPs were significantly more cytotoxic (*p* < 0.0001) by day 11 compared to day 1, which is due to their slow release of IRH over the 11 days. However, there was no significant difference in the cytotoxicity of the high-dose NPs at day 1 and day 11, which may be due to the formation of a layer of NPs on top of the cells, preventing the release and uptake of the IRH over the 11 days.

## 5. Conclusions

Shielding drugs within NPs has the potential to improve how we treat brain tumors. PCL is a flexible polymer that has been often overlooked for the manufacture of NPs due to its poor EE. However, its slow degradation and ability to provide sustained release over a longer period of time when compared to PLGA makes investigating its use for manufacturing NPs worthwhile. This research has covered a wide range of parameters and demonstrated that, through the use of amorphous PCL and the double emulsion technique, it is possible to encapsulate IRH into PCL NPs. The addition of electrolytes to both aqueous phases during the double emulsion process offers an additional benefit in encapsulating IRH by reducing its partitioning into the aqueous phase due to these phases being saturated with electrolytes. Furthermore, the IRH-loaded PCL NPs had an excellent morphology, provided zero-order sustained release of IRH for at least 16 days and are cytotoxic to primary HGGs cells over an 11-day period. This research demonstrates that IRH-loaded PCL NPs have the potential to be cytotoxic against primary HGG cells and their development should be further investigated.

## Figures and Tables

**Table 1:** Single emulsion process and formulation parameters using PVA (MW 13K) and PVP (MW 40K) surfactants.

Form. No.	pH	Sonication Time (Minutes)	Sonication Amplitude (%)	PCL Amount (mg)	IRH Amount (mg)	Surfactant Type	Surfactant%
SE 1		10	60	60	2	PVA	4%
SE 2		15	60	60	2	PVA	4%
SE 3		20	60	60	2	PVA	4%
SE 4		10	60	120	2	PVA	4%
SE 5		10	60	240	2	PVA	4%
SE 6		10	60	60	6	PVA	4%
SE 7		10	60	60	18	PVA	4%
SE 8		10	60	108	6	PVA	4%
SE 9		10	50	108	6	PVA	4%
SE 10		10	50	60	2	PVA	8%
SE 11		10	50	60	2	PVA	0.4%
SE 12		10	50	60	2	PVP	4%
SE 13	3	10	50	60	2	PVA	4%
SE 14	3	10	50	60	2	PVA	8%

**Table 2:** Double emulsion parameters of optimized formulations.

Form No.	pH	PCL Amount (mg)	IRH Amount (mg)	Aqueous Phase Volume (mL)	Surfactant Type	Surfactant %	Surfactant Average MW (g/mol)	Evaporation Time (hours)
DE 1		108	12	50	Span-80	0.3%	N/A	4
DE 2		108	6	25	PVA	4%	13K	4
DE 3		108	6	100	Span-80/Tween-80	1% Span-80 1.25% Tween-80	N/A	4
DE 4		108	6	25	PVA	4%	13K	4
DE 5		108	6	25	PVA	4%	13K	4
DE 6		108	6	25	PVA	4%	13K	4
DE 7		108	4	25	PVA	4%	13K	4
DE 8		108	6	25	PVA	4%	13K	7
DE 9		108	6	25	PVA	4%	13K	12
DE 10		108	6	25	PVA	4%	13K	24
DE 11		108	6	25	PVA	4%	89K	4
DE 12		108	6	25	PVA	8%	89K	4
DE 13		108	6	25	Span-80/Tween-20	4%	N/A	4
DE 14		108	6	25	Span-80/PVA	4%	N/A	4
DE 15		108	6	15	PVA	4%	13K	4
DE 16		108	6	50	PVA	4%	13K	4
DE 17	3	108	6	25	PVA	4%	13K	4
DE 18	3	108	6	25	PVA	4%	13K	4

**Table 3:** Summary of EE% and the influencing parameters.

Formulation	EE%	Influencing Parameter
SE9	7.5	Emulsification Method (O/W)
SE16	40.5	Emulsification Method (O/O)
SE17	12.1	Amorphization
DE1	11.1	Emulsification Method (W/O/O)
DE2	24.6	Emulsification Method (W/O/W)
DE3	18.7	Inclusion of Matrix Material
DE4	34.8	Amorphization
DE5	65.0	Amorphization/Electrolyte Addition
DE6	23.6	Amorphization/Electrolyte Addition
DE7	56.0	Drug amount
DE8	69.2	Solvent evaporation time
DE11	66.1	Surfactant MW and concentration
DE12	73.2	Surfactant MW and concentration
DE13	31.2	Surfactant Type
DE14	37.5	Surfactant Type
DE15	67.3	Aqueous Phase volume
DE16	73.7	Aqueous Phase volume
DE17	23.6	Aqueous Phase pH
DE18	22.3	Aqueous Phase pH
DE19	29.0	Surfactant concentration and pH
DE20	43.3	Surfactant concentration and pH

**Table 4:** The kinetic parameters from the different release models used for the optimized DE5 NPs.

Zero-Order		Higuchi		Korsmeyer–Peppas		
R^2^	k (% h^−1^)	R^2^	k (%h^−0.5^)	R^2^	k (%h^−n^)	n
0.9991	0.1071	0.9595	0.5093	0.9958	0.5269	0.6894

## Data Availability

The datasets and materials used and analyzed during the current study are available from the corresponding author upon reasonable request.
